# Differences in Vestibular-Evoked Myogenic Potential Responses by Using Cochlear Implant and Otolith Organ Direct Stimulation

**DOI:** 10.3389/fneur.2021.663803

**Published:** 2021-05-25

**Authors:** Isaura Rodriguez Montesdeoca, Angel Ramos de Miguel, Juan Carlos Falcon González, Silvia Borkoski Barreiro, Nicolás Pérez Fernández, Robby Vanspauwen, Angel Ramos-Macias

**Affiliations:** ^1^Department of Otolaryngology, Head and Neck Surgery, Complejo Hospitalario Universitario Insular Materno Infantil de Gran Canaria, Las Palmas, Spain; ^2^Hearing and Balance Laboratory, Las Palmas de Gran Canaria University (SIANI), Las Palmas, Spain; ^3^Otorhinolaryngology Department, Clinica Universidad de Navarra, Pamplona, Spain; ^4^European Institute for Otorhinolaryngology Head and Neck Surgery, Gasthuiszusters Antwerpen Hospitals Antwerp, Wilrijk, Belgium

**Keywords:** electrical stimulation, vestibular implant, balance, bilateral vestibulopathy, vestibulo-collic reflex, CVEMPs

## Abstract

**Objective:** Several studies have demonstrated the possibility to obtain vestibular potentials elicited with electrical stimulation from cochlear and vestibular implants. The objective of this study is to analyze the vestibular-evoked myogenic potentials (VEMPs) obtained from patients implanted with cochlear and vestibulo-cochlear implant.

**Material and Methods:** We compared two groups: in the first group, four cochlear implant (CI) recipients with present acoustic cVEMPs before CI surgery were included. In the second group, three patients with bilaterally absent cVEMPs and bilateral vestibular dysfunction were selected. The latter group received a unilateral cochleo-vestibular implant. We analyze the electrically elicited cVEMPs in all patients after stimulation with cochlear and vestibular electrode array stimulation.

**Results:** We present the results obtained post-operatively in both groups. All patients (100%) with direct electrical vestibular stimulation *via* the vestibular electrode array had present cVEMPs. The P1 and N1 latencies were 11.33–13.6 ms and 18.3–21 ms, respectively. In CI patients, electrical cVEMPs were present only in one of the four subjects (25%) with cochlear implant (“cross”) stimulation, and P1 and N1 latencies were 9.67 and 16.33, respectively. In these patients, the responses present shorter latencies than those observed acoustically.

**Conclusions:** Electrically evoked cVEMPs can be present after cochlear and vestibular stimulation and suggest stimulation of vestibular elements, although clinical effect must be further studied.

## Introduction

Vestibular system is essential for the sense of balance. It contributes to gaze stabilization through the action of the vestibulo-ocular reflex (VOR) and head stabilization in space through the activation of the neck musculature and control of posture through the vestibulocollic reflex (VCR) and the related cervicocollic reflex (CCR) complementing the VOR (1).

In bilateral vestibulopathy (BVP), patients' susceptibility for falling increases, with a higher risk of accidental injury or even death (2). Thus, for this kind of patients, therapeutic management becomes complicated because there is no effective treatment able to restore vestibular function (3–5). Vestibular rehabilitation and galvanic stimulation have been used and have a positive functional impact for these patients (6–8). In view of the foregoing, the vestibular implant represents a new vestibular rehabilitation tool with promising results (9).

Vestibular implants are based on electrical stimulation principles that were described for the first time by Suzuki and Cohen, pioneers in the electrical stimulation of the vestibular nerve branches (10). As indicated in previous studies, there are several ways of vestibular electrical stimulation that are under investigation. In our present research, we will analyze two of them: vestibular cross-stimulation using a cochlear implant (CI) and direct vestibular stimulation using a vestibular implant.

In the case of costimulation, it has been observed that the effects on the vestibular portion of the inner ear remain unclear. Histopathological studies of cadaveric temporal bones after CI demonstrated vestibular damage. Fibrosis of the vestibule and distortion of the saccular membrane have been observed (11). On the other side, reports of improved balance function after cochlear implant activation suggest that CI also have a positive impact on the vestibular system. There are also evidence that suggest that peripheral vestibular afferents are preserved after CI, even after end organ trauma (12). As stimulation current can widely spread from an intracochlear electrode array to the facial nerve, the possibility of a vestibular cross-stimulation must be also considered (13). Vestibular-evoked myogenic potentials (VEMPs) first reported by Colebatch and Halmagyi (14) are electromyographic responses from the vestibule evoked by sound, vibration, or electrical stimulation. In our study, we will reach conclusions largely based on this test results that analyze the otolith organs. Saccule and utricle constitute the otolith organs, which are sensors of linear acceleration and related reflex pathways.

On the other hand, direct electrical stimulation by vestibular implant has aimed to restore vestibular function as a whole; until now, the research has focused mainly on the restoration of the vestibulo-ocular reflex. However, recent studies have begun to evaluate the effect in the vestibulocollic and vestibulospinal reflexes (15, 16).

The objective of our study is to verify if the vestibulocollic reflex (VCR) may be evoked by electrical stimulation through a cochlear and vestibular implant. For this purpose, the EcVEMPs of the patients after implantation were analyzed.

## Materials and Methods

### Prospective, Observational, and Descriptive Study

Seven patients were included in this study between May 2019 and December 2019, divided in two groups: the cochlear implant group and the vestibular implant group. In the cochlear implant group, four patients presented severe hearing loss. In all cases, acoustic VEMP responses were present before cochlear implant implantation. In the vestibular implant group, three patients had bilateral vestibular dysfunction (BVD) and met the inclusion criteria for vestibular implantation research, which have been described in detail in a previous study (17). All three of these patients also had severe hearing loss. All patients were selected and implanted by the same surgical team (Table 1).

**Table 1:** Clinical data of both vestibular and cochlear implanted patients (group 1: cochleo-vestibular implant; group 2: cochlear implant).

**Subject**	**Age of implantation**	**Etiology**	**Implantation (year)**	**Implanted side**	**Onset**	**Sex**	**DHI Pre**	**DHI Post**	**VHIT Post**	**PTA Pre**	**PTA Post (1 year follow-up)**
**Group 1**											
VI/CI1	46	Meningitis	2018	OI	45 (2017)	Male	80	20	–	–	–
VI/CI2	41	Meningitis	2018	OI	29 (2006)	Male	28	2	–	–	–
VI/CI3	53	Meningitis	2019	OD	52 (2018)	Male	20	16	–	–	–
**Group 2**											
C1	43	Otosclerosis	2018	OI	(30) 2005	Female	6	8	N	Residual hearing	–
C2	56	Otosclerosis	2018	OI	(26) 1990	Female	0	0	N	Residual hearing	Residual hearing
C3	51	Unknown	2017	OD	(5) 1971	Female	36	34	N	Residual hearing	–
C4	54	Unknown	2020	OI	(5) 1971	Female	34	78	N	Residual hearing	–

#### Cochlear Implant Group (Four Patients)

Two patients received a CI532® implant (perimodiolar) (one case unilateral CI and one case bilateral CI). One of those cases preserved residual hearing after surgery. One case received a CI512® (straight electrode array). The surgical technique was standardized including electrode round window approach in all cases (Table 2).

**Table 2:** Characteristic of cervical VEMPs pre-operative and evoked by electrical stimulation after surgery in both groups.

**Subject**	**Electrode type**	**N–P amplitude (μV) pre**	**P1 latency (ms) pre**	**N1 latency (ms) pre**	**N–P amplitude (μV) post**	**P1 latency(ms) post**	**N1 latency(ms) post**
C1V1	CI532®/Cochlear 24RE ST	–	–	–	25.8	12.6	18.6
C2V2	CI532®/Cochlear 24RE ST	–	–	–	47.3	13.6	21
C3V3	CI532®/Cochlear 24RE ST	–	–	–	38.6	11.33	18.33
C1	IC 512	47.5	18.33	25.33	–	–	–
C2	IC 532	58.5	16.3	24.3	72.69	9.67	16.3
C3	IC532	36	18	25	–	–	–
C4	IC 532	37	15	23	–	–	–

#### Vestibular Implant Group (Three Patients)

Three patients with BVL received a new research vestibular implant (VI). The VI is a custom-modified cochlear implant with a full-band straight electrode, CI24RE (ST), from Cochlear Ltd (Lane Cove, NSW, Australia) with three active electrodes for VI stimulation (17). Full-band electrodes were selected to assure that the electrodes were facing the closest area of neural tissue related to the saccular area. For the cochlear stimulation, a Cochlear CI532® perimodiolar electrode array (Cochlear Ltd., Sydney, NSW, Australia) was used in all of them (Table 2).

#### VEMP Testing

All patients underwent cVEMP recordings, before and after surgery. In order to obtain EcVEMP recordings after surgery, a second test using Cochlear's Custom Sound Evoked Potential Software tool (version 5.2) was used. In this study, cervical vestibular-evoked myogenic potentials were obtained by using Eclipse EP 15/EP25/VEMPs (Interacoustics AS, Assens, Denmark system). In order to determine the accuracy of the calibration method, the active electromyogram (EMG) electrode was placed over on the upper third to midpoint of the sternocleidomastoid (SCM) muscle; a reference electrode was placed on the sternum, and the ground electrode was placed on the forehead. The sitting patients were instructed to turn the head >45° to the contralateral side, in order to achieve the maximum sternocleidomastoid contraction, to generate a constant tonic tension of the SCM during the recording.

The cVEMP (in response to acoustic stimulation) and EcVEMP (in response to electrical stimulation) waveform, respectively, were recorded on the ipsilateral SCM of the ear being tested. Impedance was kept below 5 kΩ. EMG signals were bandpass filtered (1–3,000 Hz) and recorded in a 25–50-ms window relative to stimulus onset. No online artifact rejection was used. For all VEMP tests, at least three trials (100 sweeps each) were conducted. We considered EcVEMPs as present when the first positive P1 peak and negative N1 peak were visible and reproducible with a peak-to-peak amplitude >20 μv (13). We established absent VEMPs if we did not obtain recognizable waveforms. When such responses were not identified after two trials, testing was ended. All registries were made at least 1 year after implantation to assess long-term responses.

#### Acoustic Stimulus

Myogenic responses were elicited by 500-Hz tone bursts (2:2:2) at a repetition frequency of 5.1/s with an intensity at 95 and 100 dB HL, delivered through calibrated headphones. The analysis time was 100 ms; the electromyographic signal was bandpass filtered from 10 to 750 Hz. Every set of 150 stimuli was averaged and repeated twice to verify the reproducibility of the response. Acoustic stimulus was used in order to analyze the possible differences between acoustic and direct electrical stimulation.

#### Electrical Stimulus

The EcVEMPs were analyzed in the cochlear implant group with the Nucleus Freedom processor (Cochlear Corp., Sydney, Australia), which delivered an electrical stimulus directly to the participant's cochlear implant, using Custom Sound EP software (Cochlear Corp.), by using a trigger system in all CI patients. Electrical stimulus was monopolar, and the base parameters are presented in Table 3.

**Table 3:** Parameters used in vestibular cross stimulation with cochlear implant.

**Type**	**Current level**	**Stimulus pulse width (μs)**	**Stimulus interphase gap (μs)**	**Stimulus NR pulse per burst (μs)**	**Stimulus duration (μs)**	**Stimulus repetition rate (Hz)**	**Number of sweeps**
**Cross-stimulation**							
MP1	180	25	7	1	57	35	1,200

The EcVEMPs in the vestibular implant group were analyzed with the processor CP910 Nucleus® 6 (Cochlear Corp., Sydney, Australia), which delivered an electrical stimulus directly to the participant's cochlear implant, using Custom Sound EP software V.6.0 (Cochlear Corp.). The EcVEMP tests were first conducted by using electrode 1 and then were repeated by using the other inserted electrodes 2 and 3 of the vestibular implant array (pulse, 50; measurement windows, 1,600 μs). All cochlear electrodes were switched off during the registry in the case of patients with vestibular implants. Monopolar stimulation (MP) MP1 + MP2 was used with trigger system, and the stimulus characteristics in every patient are explained in the next table (Table 4).

**Table 4:** Characteristics of the dynamic range of each of the patients in group 1 (vestibular implant).

**Patient**	**Dynamic range**	**Stimulus pulse width (μs)**	**C level**	**T level**	**Maxima**	**Canal frequency (Hz)**
**Direct stimulation**						
C1V1	1	25	139	138	8	900
C2V2	1	25	192	191	8	900
C3V3	1	25	196	195	8	900

The setup consists of a lap computer, cochlear POD interface, Nucleus Chronic Electrical Stimulation of the Otolith Organ Freedom processor (Cochlear Corp., Sydney, NSW, Australia), and CI24RE (ST).

We also measured horizontal angular VOR gain by vestibular head impulse test (VHIT) (ICS Impulse type 1085 from GN Otometrics A/S).

This study was conducted in accordance with the guidelines contained in the Declaration of Helsinki on Ethical Principles for Medical Research Involving Human Subjects. This work was approved by the Provincial Ethic Committee of our hospital (Id:CEIm-CHUIMI-2017/956).

## Results

The results were carried out 1 year after surgery in both patient groups: group 1, vestibular implant and group 2, cochlear implant. Six adults participated, three male and three female, age ranging from 41 to 56 years, with five unilateral implanted and one CI bilateral implanted. The hearing loss etiology was heterogeneous (Table 1). One of the implanted patient underwent her second implant 3 years after the first surgery, so we studied the two ears independently (C3–C4).

In the cochlear implant group, acoustic cVEMPs latencies before surgery were P1 from 15 to 18.33 ms and N1 from 23 to 25.33 ms, respectively. After surgery, acoustic cVEMPs were obtained in only one of the ear tested acoustic VEMPS, patient “C2” with P1–N1 latencies of 13.7 and 21 ms, respectively. It must also be mentioned that this patient preserved some residual hearing in low frequency hearing [PTA_(0.125−0.5*kHz*)_ ≤ 70 dB HL].

In the cochlear implant group, electrical cVEMPs were obtained in the same patient (C2) with latencies P1 9.67 and N1 16.33 ms and only present in basal and medium stimulation of the electrode array. Patients did not report vestibular dysfunctions during the registration. However, in one of these patients, there was a transitory worsening in balance with cochlear implant use (C2). A second cochlear implant patient (C4) presented a severe handicap after surgery, which was partially restored. We must take into account that, in this case, the patient underwent bilateral cochlear implant, and the worsening in balance appeared after the second cochlear implantation.

In our three BVD patients, acoustic cVEMPs were absent before surgery, and electrical cVEMPs were obtained in the implanted side after VI surgery. P1 and N1 latencies were 11.33–13.6 and 18.33–21 ms, respectively. These results were present 12 months after implantation, representing the activation of the vestibulocollic reflex and, consequently, of the otolith organ activation (17). We consider it interesting to note that in patients with vestibular implants, fast saturation occurs after generating a greater intensity above the threshold used in their daily use. EcVEMPs are similar to acoustic ones, and we consider not to take into account amplitude differences between electrically and acoustically evoked responses since they depend on muscle contraction (Table 5).

**Table 5:** Waveforms of cervical VEMPs (cVEMPs) in response to acoustic (left) and electrical stimulation (right).

cVEMPS acoustical pre-operative (C1, C3, C4)	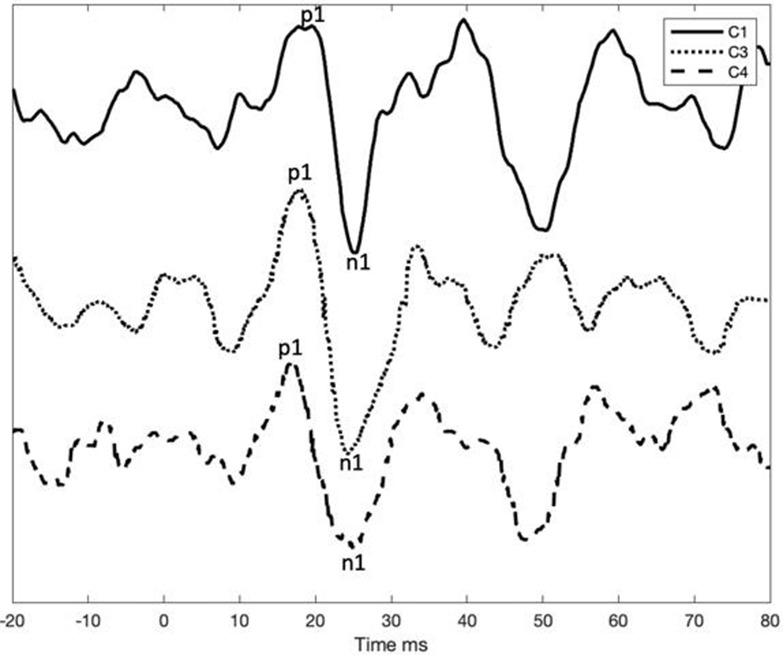
cVEMPS acoustical pre-operative and cVEMPS acoustical post-operative and ecVEMPs post-operative C2	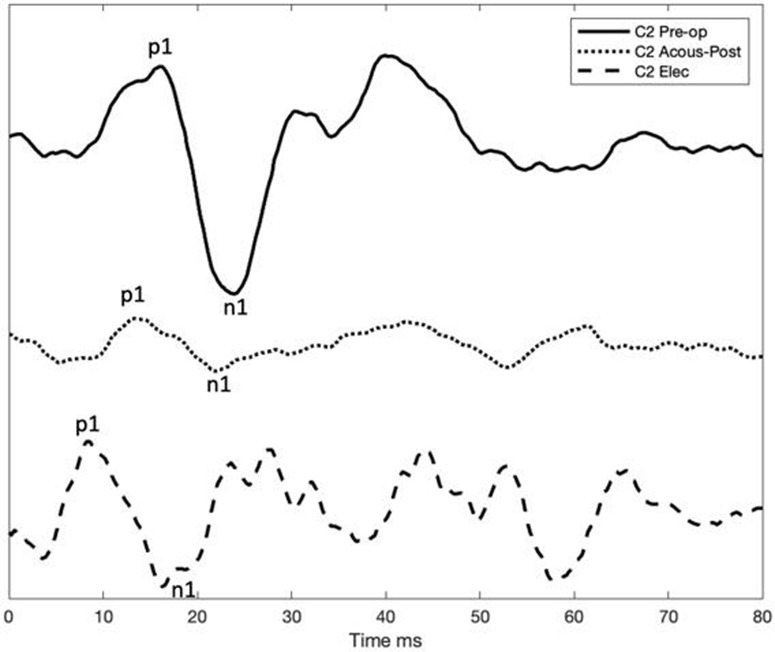
ecVEMPs post-operative C1V1, C2V2, C3V3	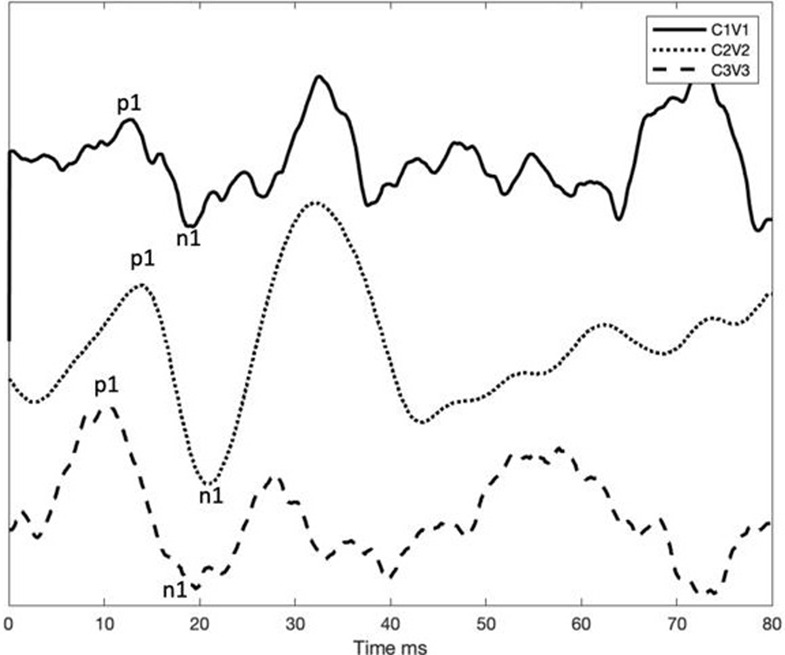

*VEMPs, vestibular evoked myogenic potentials*.

*VEMP test findings for case 3. The figures indicate the cVEMP data obtained on the side of cochlear (C1–C4) and vestibular (C1V1–C3V3) implantation (mean values)*.

VHIT analysis was performed before and after the intervention in all the subjects in the six canals. In the group of cochlear implant, VOR gain thresholds were normal (defined as >0.8), without alterations after surgery that shows persistence of the vestibular function corresponding to the semicircular canals. In BVL patients, VOR gains <0.66, before and after surgery, were found without changes in all subjects (Table 6).

**Table 6:** An example of VOR gain in six canals (VHIT) before and after cochlear implant group (example of C1 patient).

**Subject**	**VHIT Pre**	**VHIT Post**
C1	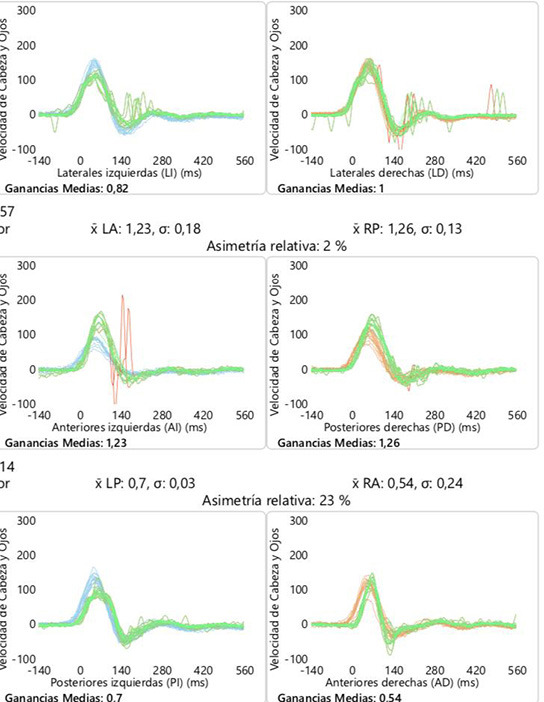	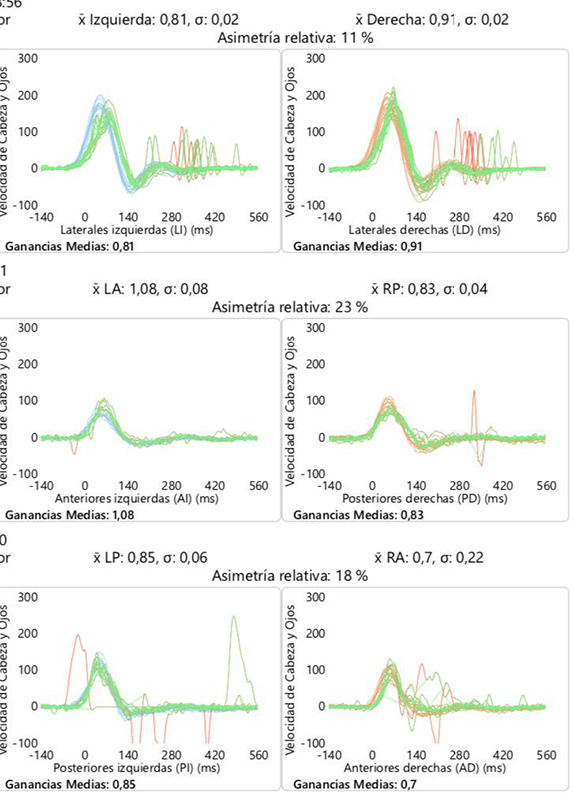

## Discussion

### Cochlear Implant

One of the objectives of this research was to analyze the costimulation effect by cochlear implant, in patients with no previous vestibular damage. One of our aims in this study was to verify if saccular function persists, taking into account the possible risk of cochlear damage and also the possible “cross” activation, by electrical stimulation with cochlear implant.

According to previous studies, the incidence of potential vestibular damage after intervention varies between 39 and 74% (18) due to trauma caused by insertion that provokes loss of perilymph (19), labyrinthitis because of foreign body reaction (20), perilymph fistula (21), and endolymphatic hydrops (22). Electrode insertion by round window cochlear implant approach has been proposed to reduce trauma to the cochlea (23–25).

Our results in the cochlear implant group show that only in one case (with some residual hearing) acoustic VEMPs were preserved. Additionally, it is confirmed that such a damage occurs in three out of four ears since acoustical cVEMPs were absent (26). Tien and Linthicum reported that 75% of the temporal bones evaluated with saccular damage coincided with damage to the basal turn of the cochlea (11). For this reason, the reduction in cochlear damage during surgery would foreseeably suppose greater preservation of hearing and saccular function, observing minor changes in post-operative VEMPS.

Furthermore, this damage was severe enough not to be reversed by costimulation of the cochlear implant stimulation. However, semicircular canals functioning remained stable in these patients, so there is no evidence of imbalance observed in the subject C3–C4 related to semicircular canals. This has also been described by Shute et al. (27) during early post-operative situation, and we observe the same situation after 1 year follow-up. In contrast, other studies show an involvement of the horizontal semicircular canal with a functional deficit in 44% of patients (18). These results also show the importance of the otolithic organs in the severity of bilateral vestibular dysfunction, which, up to now, is not included within the criteria of this clinical situation (28).

The costimulation effect has an anatomical proof/justification that has been exposed in previous studies. Current spread to the vestibular system is likely due to the effect that membranous labyrinths of the auditory and vestibular systems are connected through the fluid-filled ductus reuniens (29). In fact, vestibular and balance function can improve after CI activation in some cases (13, 30, 31).

Until now, it is unknown where the vestibular activation occurs, although due to the shortening of latencies, direct stimulation to the afferents may occur. This situation is comparable to cochlear implant stimulation of the cochlear nerve; electric current was seemingly able to bypass dysfunctional otoliths to more directly stimulate the vestibular neural/afferent elements. These observations would explain that the EcVEMPs were faster in the onset than in acoustically evoked VEMPs and comparable to the responses obtained with direct promontory stimulation (13, 17). Although there is a variability in the response, we should also take into account the influence of deafness etiology or the specific environment surrounding the electrode.

As previously described, vestibular costimulation may help to restore damaged vestibular function (17, 32) but may not be considered for all situations in cochlear implant recipients and may be related to end organ preservation after CI. Parkes et al. describe that 48% of the 96 ears studied in their study presented EcVEMPs, and in 27% of these, even without acoustic responses. Previous studies have analyzed VEMPs in children and young adults (13), but with adult samples, like the present study (>41 years old), age must be taken into account as a possible risk factor (18). For this reason, in the cochlear implant group, only patients with VEMPs prior to the intervention were chosen. However, we could not probe if age itself is decisive in costimulation. Therefore, it is necessary to obtain a greater number of cases to establish more conclusive results. The next studies should be aimed at defining what factors could be correlated with these findings: etiology, age, residual hearing, or the type of stimulation used.

### Vestibular

Electrical stimulation induces myogenic responses in the vestibulocollic pathway as has already been established before (15). We observe that otolith organ electrical stimulation can restore the vestibulocollic reflex in patients with BVD and vestibular implant, in all cases in this study, with an important effect on the clinical situation and BVD symptoms restoration (17).

The shape of the EcVEMP was similar to the conventional acoustically elicited cVEMP. However, the latencies were shorter, similar to previous observations in studies comparing galvanic stimulation (33) and electrical stimulation by cochlear implant (13). We hypothesize whether the latency variation of the response in different studies could be explained because of different circumstances: vestibular implant location, implant design, and differences in the stimulation profile and vestibular etiology, which could explain the differences found.

We found an increase in amplitude in a very short range of increasing intensity, observing a quick saturation in the response. Our findings are not directly comparable with other groups (15). The differences could be related to the kind of stimulus or semicircular canal contribution in this reflex during the semicircular canal stimulation. It is suggested that convergent neurons may receive both canal and otolith stimulation that contribute to the vestibulocollic reflex, but this circumstance is reduced in the VOR (34). This supports the idea that the selective reflexes can be elicited from different end organs (35, 36). Other options that must be under consideration is the etiology of these dysfunctions, since in our three patients, meningitis was the etiology; other groups included traumatic or genetic origin (DFNA9).

The stimulus used to evoke EcVEMPs were not perceived by the cochlear group tested; however, in the second group, they perceived an immediate stability sensation without unpleasant sensation, which implies an improvement in their clinical situation. This soft sensation must be considered, as the vestibulocollic pathway shows a low threshold of stimulation than other pathways such as the vestibulo-ocular reflex. In our case, we are not able to obtain an improvement in VOR gain by VHIT, so it might imply that there is a selective stimulation of vestibular afferents and different vestibular pathways have different activation profiles (37). However, we are not able to explain how this inputs are “processed” by the central system.

In functional conditions, our BVD patients recovered a good quality of life, with improvement in their stability, and activities in their daily life remained stable during chronic implant use for more than a year, as it has been presented previously (17). Aside from VEMPs being anticipated in future articles, there are other objective responses in vestibular implant sample such as subjective visual vertical (SVV), subjective visual horizontal (SHV), and dynamic visual acuity (DVA), which justify the restoration of vestibular function.

This results can be explained or discussed in light of an otolith selective response, given the shortening of latencies, or this can be also explained by a current spread or central convergence of the primary vestibular afferents on the second-order vestibular nuclei neurons as has been theorized in other studies (32, 38–41).

Although we have observed in this study shortened latencies in both stimuli, we did not observe a constant response due to costimulation in all patients. In these cases, vestibular activation seems to be present only when residual hearing is preserved (less iatrogenic damage) and in patients who previously presented vestibular function. Although in this study the number of patients is very small, we may assume that costimulation would be possible if the saccular damage is not severe. However, in the case of direct stimulation, we can evoke responses in previously areflexic patients; therefore, it should be considered in severe cases.

Given the small number of previous studies on chronic electrical stimulation, future challenge to obtain the maximum benefit for patients should be aimed to:

1. define the involvement of the otolith organs and semicircular canals in the vestibulocollic reflex;
2. analyze if new parameters in the electrical stimulation and vestibular prothesis design would obtain a selective activation of different reflexes;
3. achieve better EcVEMPs understanding through a larger sample of patients implanted with vestibular prostheses;
4. define effects, incidence, and possible risk factors of otolith function damage after cochlear implant and the underestimated presence of vestibular cross-stimulation.

#### Weakness

This study had some limitations. First of all is the very small sample that did not allow us to provide statistical analyses (in this phase of the research, only a very limited number of patients can be included in this research). Second, it is difficult to make comparisons in the electrical response, since it is the first vestibular implant with the otolithic organs chronic stimulation used in humans.

#### Conclusion

Electrically cVEMPs may be present after 12 months of follow-up of chronic vestibular stimulation mainly in patients with vestibular implant and a small number of patients with cochlear implant. This suggest that stimulation of vestibular elements is feasible, although the clinical effects must be further studied.

## Data Availability Statement

The raw data supporting the conclusions of this article will be made available by the authors, without undue reservation.

## Ethics Statement

The studies involving human participants were reviewed and approved by Comite etico—Complejo Hospitalario Universitario Insular Materno Infantil. The patients/participants provided their written informed consent to participate in this study.

## Author Contributions

IR: data acquisition and manuscript writing. AR: manuscript writing and graphics. JG: data acquisition and patients fitting. SB: manuscript. NP and RV: verification of VEMPS recordings. AR-M: original idea, manuscript, and verification. All authors contributed to the article and approved the submitted version.

## Conflict of Interest

The authors declare that the research was conducted in the absence of any commercial or financial relationships that could be construed as a potential conflict of interest.

## Abbreviations

BVP: bilateral vestibulopathy
CCR: cervicocollic reflex
cVEMP: cervical-vestibular evoked myogenic potentials
DVA: dynamic visual acuity
EcVEMPs: electrical cervical vestibular-evoked myogenic potentials
CI: cochlear implant
SHV: subjective visual horizontal
SVV: subjective visual vertical
VCR: vestibulocollic reflex
VI: vestibular implant
VEMPs: vestibular-evoked myogenic potentials
VHIT: vestibular head impulse test
VOR: vestibulo-ocular reflex.
